# Serum procalcitonin level and leukocyte antisedimentation rate as early predictors of respiratory dysfunction after oesophageal tumour resection

**DOI:** 10.1186/cc4992

**Published:** 2006-07-19

**Authors:** Lajos Bogar, Zsolt Molnar, Piroska Tarsoly, Peter Kenyeres, Sandor Marton

**Affiliations:** 1 Department of Anaesthesiology and Intensive Care, University of Pecs, Hungary

## Abstract

**Introduction:**

Postoperative care after oesophageal tumour resection holds a high risk of respiratory complications. We therefore aimed to determine the value of systemic inflammatory markers in predicting arterial hypoxaemia as the earliest sign of developing lung injury after oesophageal tumour resection.

**Methods:**

In a prospective observational study, 33 consecutive patients were observed for three days (T1–T3) after admission (T0) to an intensive care unit following oesophageal tumour resection. The daily highest values of the heart rate, axillary temperature, leukocyte count and PaCO_2 _were recorded. Serum C-reactive protein and procalcitonin concentrations and the leukocyte antisedimentation rate (LAR) were determined at T1 and T2. Respiratory function was monitored 6-hourly measurement of the PaO_2_/FIO_2 _ratio, and the lowest value was recorded at T3. Patients were categorised as normoxaemic or hypoxaemic using the cutoff value of 300 mmHg for PaO_2_/FIO_2_.

**Results:**

Seventeen out of 33 patients were classified as hypoxaemic and 16 patients as normoxaemic at T3. Increases of temperature at T0 and of the procalcitonin and LAR values at T2 were predictive of hypoxaemia at T3 (*P *< 0.05, *P *< 0.01 and *P *< 0.001, respectively). The area under the receiver-operating characteristic curve was 0.65 for the temperature at T0, which was significantly lower than that for the procalcitonin level at T2 (0.83; 95% confidence interval, 0.69–0.97; *P *< 0.01) and that for LAR at T2 (0.89; 95% confidence interval, 0.77–1.00; *P *< 0.001).

**Conclusion:**

These results suggest that an elevated LAR (>15%) and an elevated procalcitonin concentration (>2.5 ng/ml) measured on the second postoperative day can predict next-day arterial hypoxaemia (PaO_2_/FIO_2 _< 300 mmHg) after oesophageal tumour resection.

## Introduction

Oesophageal tumour resections carry a considerable risk of early postoperative complications. The consequent inhospital mortality rate can be as high as 10–15% [1]. Atelectasis formation has been identified as a leading cause of early secondary morbidity after oesophagectomy [2]. The preceding clinical signs that can be linked to the atelectasis formation and consequent arterial hypoxaemia, however, have not been studied after oesophagectomy.

Our group previously observed that procalcitonin (PCT) as a marker of the severity of bacterial infections failed to predict postoperative inflammatory complications after major operations [3]. On the contrary, Brunkhorst and colleagues found PCT a reliable marker in discrimination of infectious and noninfectious causes of early acute respiratory distress syndrome [4]. The link between a surgical insult and the subsequent lung injury seems obvious and lies among the inflammatory processes mediated by the interaction of neutrophil leukocytes, endothelial cells and epithelial cells of the lung.

We previously reported a leukocyte function test measuring the number of upward floating (that is to say, antisedimenting) leukocytes in a sedimentation tube during one hour of gravity sedimentation [5]. The leukocyte antisedimentation rate (LAR) indicates the percentage of leukocytes crossing the middle line of the blood column upwards during 1 hour of sedimentation. It has been proven that an elevated LAR is in positive correlation with enhanced leukocyte adherence [6], with an increased cell volume and higher vacuole content of neutrophil leukocytes [7], and with the severity of systemic inflammatory reaction syndrome (SIRS) in critically ill patients [5].

The aim of this study was to investigate whether conventional and newly developed inflammatory markers (SIRS components, C-reactive protein (CRP), PCT and LAR) measured on the first and second postoperative days of oesophageal tumour resections could predict third-day arterial hypoxaemia as the earliest sign of evolving respiratory dysfunction. The study intended to test inflammatory markers that have been taken up by the intensive care practice and a relatively new tool that is easy to use (LAR).

## Materials and methods

Following local ethics committee approval, informed consent was obtained from 33 consecutive patients (Table 1) who entered our prospective observational study after admission to our eight-bed teaching hospital intensive care unit (ICU) following elective oesophageal tumour resection. All operations were performed by the same two surgeons via a transthoracic or transhiatal approach as appropriate. Intraoperative heat loss was reduced using forced-air heating blankets applied on skin surfaces of the patient not involved in the surgical exploration. All patients were admitted to the ICU awake with spontaneous breathing after extubation. Single-shot surgical antibiotic prophylaxis was administered in all patients. Postoperative analgesia was provided via thoracic epidural canula for all of our patients, and the level of pain sensation was kept below three on the visual analogue scale in the entire observational period. The axillary temperature was measured four-hourly after admission to the ICU (T0) and on the first and second postoperative days (T1 and T2, respectively). The heart rate and PaCO_2 _were measured one-hourly and six-hourly, respectively, and the daily highest and lowest values, respectively, were recorded. The patients' clinical progress was monitored by daily multiple organ dysfunction scores (MODS) [8]. The lowest PaO_2_/FIO_2 _ratio value was taken on postoperative day three (T3). A chest X-ray scan was taken on the first postoperative day and later when developing intrapulmonary infiltrates were suspected by considering the axillary temperature, chest auscultation and arterial blood gas analysis results.

The peripheral leukocyte count was measured eight-hourly at T1 and T2 and the highest values were recorded. There were no conflicting high and low values of the leukocyte count (<4.0 or >12.0 × 10^3^/μl) and the axillary temperature (<36.0 or >38.0°C) in the same patient on the same day. Five millilitres of arterial blood samples were drawn for measuring PCT and CRP levels into serum separator tubes at T1 and T2. Samples were immediately centrifuged, and sera were separated and stored at -70°C. The PCT concentration was measured by immunoluminometric assay (LUMItest, normal range <0.5 ng/ml; Brahms Diagnostika, Berlin, Germany). The CRP level was determined by nephelometric assay (normal range <10 mg/l; Orion Diagnostics, Helsinki, Finland).

The LAR was measured by leukocyte counting in the upper half and in the lower half of the sedimentation blood column after one-hour gravity sedimentation of the whole blood at T1 and T2 [5]. The formula LAR = 100 × (upper - lower)/(upper + lower) was then used to calculate the percentage of leukocytes that crossed the middle line of the sedimentation blood column upwards during 1 hour of sedimentation (normal range <10%). The interassay coefficient of variation for the CRP, PCT and LAR measurements was <5%.

### Statistical analysis

Patients were categorised as normoxaemic or hypoxaemic according to the lowest value of the PaO_2_/FIO_2 _ratio measured at T3 being greater than or smaller than 300 mmHg. Results are demonstrated as medians and interquartile ranges. Mann–Whitney and Fisher's exact tests were performed to assess the differences between normoxaemic or hypoxaemic patient subgroups. A Bonferroni correction was calculated for each group of comparisons. The number of patients required was calculated by power analysis according to LAR results from our previous study, performed on a similar population, in which a LAR greater by 15% (standard deviation 9%) showed a 91% sensitivity of predicting blood culture positivity [9]. With type I α = 5% and with type II (power) of 90%, we therefore needed 32 patients. The receiver-operating characteristic (ROC) curves and the areas under the respective curve were calculated. The values of SIRS components, CRP and PCT levels and the LAR measured at T1 and T2 were used to calculate the ROC curves. Statistics were performed using the Statistical Program for Social Sciences (SPSS^® ^version 10.0) software for Windows (SPSS, Chicago, Ill., USA).

## Results

There were no significant differences between the hypoxaemic group (*n *= 17) and the normoxaemic group (*n *= 16) regarding age, gender ratio, surgical approach of oesophageal resection and operation time. Hypoxaemic patients, however, stayed significantly longer in the ICU and had a higher ICU mortality rate than normoxaemic patients (*P *< 0.001 and *P *< 0.05, respectively; Table 1). Temperatures taken at T0 were significantly higher in hypoxaemic patients compared with normoxaemic patients (*P *< 0.05). The temperature, heart rate, PaCO_2_, leukocyte count, serum concentration of CRP, PaO_2_/FIO_2 _ratio and MODS score at T1 and T2 were not different between patient subgroups. The PCT level and LAR showed no statistical differences at T1, but at T2 both values were significantly elevated in the hypoxaemic group compared with the normoxaemic group (*P *< 0.01 and *P *< 0.001, respectively; Table 1). Intrapulmonary infiltrates were undetectable by chest X-ray scans in any of the patients. Similarly, no other sites of infection could be detected in the observation period of three days.

Further statistical analysis of these significant differences provided an area under the ROC curve of 0.65 (95% confidence interval (CI), 0.47–0.82) for the temperature at T0. This was significantly lower than that for the PCT level at T2 (0.85; 95% CI, 0.71–0.99; *P *< 0.01) and the LAR at T2 (0.89; 95% CI, 0.77–1.00; *P *< 0.001). The sensitivity, specificity, positive and negative predictive values, and likelihood ratios of LAR results at T2 indicate that 15% provides the best discrimination between the hypoxaemic and normoxaemic endpoints at T3 (Table 2).

## Discussion

This recent study demonstrates that an elevated LAR (>15%) and an elevated PCT level (>2.5 ng/ml) measured on the second postoperative day can predict next-day arterial hypoxaemia as one of the early signs of threatening respiratory complication after oesophageal tumour resection. The area under ROC curve that used the day two PCT concentration to detect next-day decay of oxygen uptake was 0.85 (95% CI, 0.71–0.99). This was a significantly smaller area under the ROC curve value (*P *< 0.05) than for the LAR measured at the same time point (0.89; 95% CI, 0.77–1.00). Our results suggest that LAR >15% can be a valuable marker of early postoperative respiratory insufficiency after oesophageal tumour resection. On the other hand, when focusing on day three respiratory insufficiency, the predictive values of the SIRS components, the CRP concentration, the PaO_2_/FIO_2 _ratio and the MODS score were poor.

A number of recent publications have reported an acceptable predicting power of the PCT level for the severity of infectious complications in different cohorts of intensive care patients [10-13]. Our present observation is different to these previous ones, however, because we investigated the signs that precede the conventional signs of infection, severe sepsis, septic shock or MODS. Our endpoint was the commencement of respiratory insufficiency marked by the lowest PaO_2_/FIO_2 _ratio (cutoff point, 300 mmHg) on day three. The relevance of distinguishing these hypoxaemic and normoxaemic subgroups was proven by the significantly different ICU mortality rates. Our results underline the importance of preliminary complications such as a deteriorating PaO_2_/FIO_2 _ratio because it can progress into more severe patient conditions. The main message of this recent observation is that the detection of increasing PCT and LAR values, even without signs of infection, can be regarded as a hint at forthcoming respiratory insufficiency and, later on, other complications.

Regarding the first three postoperative days, Ranieri and colleagues found that noninfectious ventilatory damage of the lungs is associated with increased intrapulmonary sequestration of neutrophils [14]. This process can be one of the consequences of leukocyte activation by circulating, soluble inflammatory mediators. We have proven that activated leukocytes exert increased cellular volume due to water uptake, resulting in a higher rate of antisedimentating leukocytes [7].

Mild intraoperative hypothermia is associated with a threefold increase in morbid myocardial events, increasing the risk of wound infection and blood loss [15]. Although we made every effort to prevent intraoperative heat loss, some of our patients (*n *= 9) recovered from surgery in slight hypothermia (T0 < 36.5°C) that was due to extensive surgical exploration resulting in decreased skin surfaces for warming blankets. It is surprising to note that the subgroups of our patients with the lowest axillary temperature at T0 presented the highest PaO_2_/FIO_2 _ratio at T3. This can be explained by the previous animal experiments stating that a subnormal core temperature can be protective against lung injury [15,16].

No intrapulmonary bilateral infiltrates were detected by chest radiography and physical examinations during this time. Nine hypoxaemic patients' respiratory insufficiency progressed after T2 and T3, however. These nine patients required mechanical ventilation, and eventually five of them died in the ICU due to bronchopneumonia, septic shock and multiple organ failure.

Limitations of the present study are the short observation period, the possible inaccuracy of taking the axillary temperature, the relatively small sample size and the lack of monitoring of other soluble inflammatory mediators. The true septic consequences would have been detected by recording further progression of patients' inflammatory reactions.

## Conclusion

Components of SIRS and the serum concentration of CRP fail to predict threatening diminishment of the PaO_2_/FIO_2 _ratio. An elevated PCT level and, especially, an elevated LAR indicate forthcoming arterial hypoxaemia in the early postoperative period of oesophageal tumour resections.

## Key messages

• Two days after oesophageal tumour resection, elevation of the serum PCT concentration can predict next-day diminishment of the PaO_2_/FIO_2 _ratio.

• On the second postoperative day, a LAR higher than 15% was also predictive in respect of third-day respiratory insufficiency.

• Patients with a PaO_2_/FIO_2 _ratio less than 300 mmHg at T3 had a higher axillary temperature on admission than patients with a PaO_2_/FIO_2 _ratio of 300 mmHg or higher.

• CRP and components of systemic inflammatory response syndrome measured at T1 and T2 failed to predict a diminished PaO_2_/FIO_2 _ratio at T3.

## Abbreviations

CI = confidence interval; CRP = C-reactive protein; ICU = intensive care unit; LAR = leukocyte antisedimentation rate; MODS = multiple organ dysfunction scores; PaCO_2 _= arterial carbon dioxide pressure; PaO_2_/FIO_2 _= arterial oxygen tension per fractional inspired oxygen concentration; PCT = procalcitonin; ROC = receiver-operating characteristic; SIRS = systemic inflammatory reaction syndrome.

## Competing interests

The authors declare that they have no competing interests.

## Authors' contributions

LB and ZM developed the study design and coordinated the manuscript preparation. PT performed data collection and manuscript preparation. PK contributed to the study design and manuscript preparation. SM was responsible for data collection and carried out the statistical analysis. All authors read and approved the final manuscript.

## Figures and Tables

**Table 1:** Patient characteristics and postoperative inflammatory markers

	T3 PaO_2_/FIO_2 _ratio <300 mmHg		T3 PaO_2_/FIO_2 _ratio ≥ 300 mmHg	
Age (years)	61 (49–66)		57 (53–56)	
Males/females (*n*)	2/15		3/13	
Transhiatal oesophagus resection/transthoracic oesophagus resection (*n*)	5/12		9/7	
Length of operation (minutes)	315 (281–360)		356 (312–395)	
ICU length of stay (days)	9 (6–18)		3.5 (3–5)***	
ICU mortality/survival (*n*)	5/12		0/16*	
Temperature at T0 (°C)	37.3 (37.0–37.6)		36.4 (36.1–37.9)*	
	
	T1	T2	T1	T2
	
Temperature (°C)	37.2 (37.0–37.5)	37.5 (36.7–37.8)	37.0 (37.0–37.3)	37.0 (36.8–37.1)
Leukocytes (×10^9^/l)	9.1 (5.7–11.0)	11.7 (6.2–14.0)	8.1 (5.8–8.8)	10.1 (7.9–11.4)
Heart rate (minute^-1^)	94 (88–98)	113 (102–119)	92 (77–94)	109 (91–119)
PaCO_2 _(mmHg)	38.2 (32.4–39.5)	39.9 (32.3–40.8)	36.4 (33.4–37.8)	36.8 (34.8–38.7)
PaO_2_/FIO_2 _ratio (mmHg)	185 (149–267)	251 (169–286)	230 (200–251)	302 (260–340)
Multiple organ dysfunction scores	3 (2–4)	3 (2–4)	2 (1–3)	2 (1–3)
C-reactive protein (mg/l)	98 (79–126)	106 (71–122)	101 (89–113)	115 (70–127)
Procalcitonin (ng/ml)	0.5 (0.3–0.7)	2.8 (0.7–5.1)	0.7 (0.6–0.9)	1.2 (1.0–2.1)**
Leukocyte antisedimentation rate (%)	4.9 (3.2–7.9)	19.2 (13.6–28.0)	9.2 (5.6–13.8)	9.7 (2.5–11.4)***

ICU, intensive care unit. Groups were compared by Fisher's exact test and the Mann–Whitney test as appropriate. Median (quartiles) and statistical differences were calculated between PaO_2_/FIO_2 _ratio values at corresponding time points. *P < 0.05, **P < 0.01, ***P < 0.001.

**Table 2:** Predictive values of the procalcitonin serum level and the leukocyte antisedimentation rate assessment at T2 for the four cutoff values that separate the five quintiles of parameter distribution

Cutoff value	Cases below the cutoff value (*n*)	Sensitivity (%)	Specificity (%)	Positive predictive value (%)	Negative predictive value (%)	Positive likelihood ratio	Negative likelihood ratio
Procalcitonin (ng/ml)							
0.69	7	27	9	62	14	0.3	8.1
1.23	13	68	55	75	46	1.5	0.6
2.43	20	52	83	85	50	3.1	0.6
4.43	26	30	100	100	38	>20	0.7
Leukocyte antisedimentation rate (%)							
6.3	7	88	31	58	71	1.7	0.2
10.6	13	76	56	65	69	1.5	0.6
12.7	20	71	94	92	75	6.1	0.5
23.2	26	41	100	100	62	>20	0.7
